# Validation of the CogState battery for rapid neurocognitive assessment in Ugandan school age children

**DOI:** 10.1186/s13034-015-0063-6

**Published:** 2015-08-14

**Authors:** Paul Bangirana, Alla Sikorskii, Bruno Giordani, Noeline Nakasujja, Michael J Boivin

**Affiliations:** Department of Psychiatry, Makerere University, Kampala, Uganda; Department of Statistics and Probability, Michigan State University, East Lansing, MI USA; Neuropsychology Section, Department of Psychiatry, University of Michigan, Ann Arbor, MI USA; Departments of Psychiatry and of Neurology and Opthalmology, Michigan State University, East Lansing, MI USA

**Keywords:** Neuropsychological assessment, Validity, Reliability, African children

## Abstract

**Background:**

CogState is a widely used computer-based cognitive test whose validity has not been addressed in resource poor settings. We examined the construct, concurrent and convergent validity of CogState, test–retest reliability and the effect of sociodemographic variables on CogState outcomes in school age children.

**Methods:**

Two hundred and thirty Ugandan children (54% male) with mean age 6.99 years (SD = 1.67, range 5–13 years) were assessed using CogState, the Kaufman Assessment Battery for Children, 2nd edition (KABC-II) and the Test of Variables of Attention (TOVA) at baseline and 8 weeks later. Correlations were run between CogState and the KABC-II and TOVA to evaluate its concurrent and convergent validity. Factor analysis was used to evaluate construct validity of CogState. Correlations between baseline and 8 weeks CogState scores were used to determine the test–retest reliability while general linear models were used to assess associations with sociodemographic factors.

**Results:**

Significant correlations were observed between CogState’s One Card Learning, One Back Memory and Card Detection with the TOVA and between CogState’s Maze Chase and One Back Memory with KABC-II’s Simultaneous Processing. CogState had a three factor structure with Processing Speed, Processing Accuracy and Maze Chase and Maze Learning. CogState had low to moderate test–retest reliability in Ugandan children with correlations ranging from 0.32 to 0.57. Age, sex and education were associated with CogState outcomes.

**Conclusions:**

CogState is a valid and reliable test battery for rapid computer-based neurocognitive assessment in Ugandan children and can thus be used in this cultural context.

**Electronic supplementary material:**

The online version of this article (doi:10.1186/s13034-015-0063-6) contains supplementary material, which is available to authorized users.

## Background

Assessment of cognition in African children has provided important insights on how infectious diseases, poverty, nutritional and social factors affect cognition [1–3]. Interventions aimed at improving the developmental potential of at risk children have also been evaluated by cognitive testing [4–8]. Despite these important applications of cognitive tests, most of them take considerable time to administer, are often translated and adapted from Western-based tests relying heavily on language, need well trained assessors and cannot be administered in the home setting [9]. This limits their utility for quick assessment and field testing. A brief comprehensive cognitive battery that can easily be administered in the field will be of great practical benefit in cognitive testing in African children. This study was designed to test the validity and reliability of CogState, a brief computer-based measure of neurocognitive ability.

CogState is a computer administered test that assesses a number of cognitive skills including reaction time, working memory, learning and attention [10]. Test scores are computer generated thus reducing the risk of human error in administration and scoring and allowing for more exact timing of response speed. It has the advantages of being portable (adaptable to notebooks, tablets, and even smart phones), short (20–30 min), game-like in presentation and thus motivating, and cross-culturally adaptable because it is language independent. In the US and Asia, CogState has been used to assess working memory and cognition in children with eating disorders and movement difficulty and the effect of a summer treatment program on cognition in children with ADHD [11–13]. It has proved to be valid and reliable in healthy children and among patients with schizophrenia, HIV/AIDS and mild traumatic brain injury [14, 15].

CogState has been used to evaluate the effect of cognitive rehabilitation programs in Ugandan children with HIV and those who survived cerebral malaria [6–8]. There is however no data from this setting attesting to the test’s validity and reliability in the sub-Saharan African cultural context, where children are often at risk from a host of factors pertaining to poverty, malnutrition, toxic environmental exposure, and infectious disease [16] and may have little experience with computer-based presentations. The primary aim of this study was to examine the concurrent and convergent validity of CogState between the Test of Variables of Attention and the Kaufman Assessment Battery for Children (second edition) which have proven sensitive to the neurocognitive effects of severe malaria and HIV in our studies in Uganda [3, 17–20]. The second aim of this study was to examine the construct validity of CogState using factor analysis. The third aim was to determine the test–retest reliability of CogState by comparing two test scores given at different times. The fourth aim examined the effect of sociodemographic variables (age, sex, child and parental education, weight, height, socioeconomic status and quality of the home environment) on CogState outcomes and to compare findings to our previous results using the KABC and TOVA where sociodemographic variables were predictive of scores in these tests [21].

## Methods

### Study participants

Study children were aged 5–13 years and had been recruited into a computerised cognitive rehabilitation training study between 2011 and 2014 at Mulago Hospital, Kampala. This intervention study from which participants for the present study were drawn had three groups of children; children with a history of cerebral malaria (CM), a history of severe malarial anemia (SMA) and health community controls (CC) who had no history of severe malaria. Those who had a history of CM or SMA on average 33 months earlier (range 23–62 months) and healthy community controls were recruited into the present study. These children were not included in the present study because of the different groups they belonged to (CM, SMA or CC), but because the testing procedures they went through provided an excellent opportunity to test the validity of CogState.

Cerebral malaria was defined as: (1) coma [Blantyre Coma Score (BCS) ≤2); (2) *Plasmodium falciparum* on blood smear; and (3) no other known cause of coma (e.g., meningitis, prolonged postictal state, or hypoglycemia-associated coma reversed by glucose infusion). Severe malarial anemia was defined as presence of *P. falciparum* on blood smear in children with a hemoglobin level ≤5 g/dL. Children with CM or SMA were managed according to the Ugandan Ministry of Health treatment guidelines current at the time of admission.

Healthy community children (CC) were recruited from the nuclear family, extended family, or household compound area of children with CM or SMA. Eligible CC were aged 5 months to 12 years. Children were enrolled if they met inclusion criteria and did not meet exclusion criteria. Exclusion criteria for all children included: (1) known chronic illness requiring medical care; (2) known developmental delay; or (3) prior history of coma, head trauma, hospitalization for malnutrition, or cerebral palsy; (4) severe neurologic complications from the malaria episode that hinder the child’s ability to comprehend instructions and perform manual tasks on the computer.

Additional exclusion criteria for children with SMA included: (1) impaired consciousness on physical exam; (2) other clinical evidence of central nervous system disease; or (3) >1 seizure in the past 24 h prior to admission. Additional exclusion criteria for CC included: (1) illness requiring medical care within the previous 4 weeks or (2) major medical or neurological abnormalities on a screening physical exam.

Written informed consent was obtained from parents or guardians of study participants and assent from children aged 7 years and older. Ethical approval was granted by the Institutional Review Boards for human studies at Makerere University School of Medicine and Michigan State University.

### Study assessments

All children, regardless of group underwent the same physical examination and provided a medical history. Other variables like parental education, quality of the home environment and socioeconomic status score were accessed from the database of another observational study in which they had participated earlier [1]. All children found to be healthy were sent for cognitive assessment using CogState, the KABC-II and the TOVA.

CogState is a brief computer administered test assessing a number of cognitive abilities. The battery used in this study uses both regular playing cards and mazes to assess cognition. The maze tasks are the *Groton Maze Chase Task* Measuring visuomotor processing speed and the *Groton Maze Learning Task* measuring spatial working memory and learning which are presented on a 10 × 10 computer grid of tiles. In the maze chase task the subject is required to follow a target moving along the grid by clicking the tiles it has passed through making sure not to skip tiles or move diagonally. The primary outcome here was the total number of correct moves made per second. In the maze learning task the subject is required to find a hidden pathway through the grid from the top left tile to the bottom right one by guessing his/her way through the grid while receiving feedback on each individual move. The primary outcome was the total number of errors made in learning the task in one to five trials in a single session.

The card tasks include *Detection* measuring psychomotor speed where the subject is required to press the YES button on the mouse as soon as a card turns face-up. *Identification* measuring visual attention where the subject is required to decide as quickly as possible whether the card that has turned face up is red (YES button) or not (NO button); *One Card Learning* measuring visual learning and memory where the subject decides whether the card has appeared before in the task by pressing the YES or NO buttons, and *One Back Memory* measuring working memory where the subject is required to immediately decide if the card is the same (YES button) as the previous one or not (NO button). For both Detection and Identification, the speed of performance (mean of the log10 transformed reaction times for correct responses) was the primary outcome while for One Card Learning and One Back Memory the accuracy of performance (arcsine transformation of the square root of the proportion of correct responses) was the primary outcome. The tests rely on minimal spoken language, which can be easily translated and include learning trials that continue until the child has consistently understood the concepts involved in the particular task.

The KABC-II has been widely used among Ugandan children to assess overall cognition and specific abilities like working memory, visual processing, reasoning and learning (long-term storage and retrieval) [3, 8, 17, 18]. It has a stable internal structure when used among Ugandan children and its scores are associated with the quality of the home environment, nutritional status and child’s level of education [21, 22]. Administration of the KABC-II is not heavily dependent on spoken language which makes the test easy to administer across cultures. However, test instructions were translated and back translated into Luganda, the commonly spoken language, which were then used in administration. The primary outcomes were short-term memory (taking in information, holding it then use it within a short time), visual processing (perceiving, manipulating and thinking visually), reasoning (solving problems by induction and deduction) and long-term storage and retrieval (storing and retrieving newly or previously learned information).

The TOVA is a computer-administered test of attention for children aged 5 years and older, as well as for adults. It also measures other facets of attention like reaction time, impulsivity, inattention and reaction time variability. The TOVA has been widely used in Ugandan children and is able to distinguish between children affected by central nervous system diseases and controls [3, 17–20]. The stimuli involved in the task are only simple geometric square shapes, familiar to most all individuals. The TOVA outcomes considered for this study were; the d’ prime score (a measure of overall attention capacity), inattention (failing to respond to the target), impulsivity (responding to the wrong target), response time (time taken to respond), response time variability (variability in the response time of correct responses) and ADHD score (similarity of the score to that of an ADHD sample). Instructions for TOVA were explained to children in Luganda for those who did not understand English. A practise session was then given before the actual testing to ensure the test was well understood. For both the KABC and TOVA, there are no local norms in Uganda and studies have therefore used control groups to compare with the exposed sample [1, 17–20].

Cognitive testing was performed on children at baseline after enrolment in the intervention study after which all three groups were randomised to three trial arms of the study. Two types of cognitive rehabilitation intervention (full and limited) were administered. A total of 77 children (33%) received the full version of the intervention, 85 (37%) received a limited version of the intervention, and 68 (30%) were controls. Repeat cognitive testing was performed 8 weeks later, after completion of the cognitive rehabilitation training intervention period. For this study, only the test results at 8 weeks of the control group that received no intervention were used. This control group did not participant in any procedures in the 8 weeks.

### Statistical analysis

Weight for age z scores and height for age z scores were calculated using CDC norms (Epi Info version 3.5.3; Centers for Disease Control and Prevention). Detection scores of children that had invalid Detection performance were omitted from the analyses. Test scores from CogState, KABC-II and TOVA were first age adjusted by converting them to z scores using the means of the community controls of the present study. This was done using the formula; (actual child’s score − mean score of that age of the CC group)/standard deviation. This method has also been used elsewhere among Ugandan children [1]. Construct validity of CogState was evaluated by running an exploratory factor analysis with promax rotation to facilitate interpretation of the resulting factors. This approach was chosen to identify the latent factors within the different tests of CogState and whether tests measuring similar constructs load on the same factor. This approach has been used in other cross-cultural validation studies of tests developed in the West [22, 23]. The concurrent and convergent validity of CogState were analysed by evaluating Pearson correlations between its scores and those from the KABC-II and TOVA. In particular, concurrent validity was evaluated using outcomes assessing the same abilities; CogState detection and TOVA response time, CogState identification and TOVA d′ prime, CogState Maze Learning and KABC-II Short-term Memory, CogState One Card Learning and KABC-II Long-term Storage and Retrieval, CogState One Back Memory and KABC-II Shot-term Memory, CogState Maze Chase and TOVA Reaction Time. Correlations between pairs of variables reflecting similar but different constructs provided evidence of convergent validity. Pearson correlations of CogState variables measured at baseline and week 8 in the control group of the trial (consisting of children from the CM, SMA and CC groups randomised to this group) were used to gauge test–retest reliability of CogState. Finally the effects of age, sex, child and parental education, weight, height, socioeconomic status, quality of the home environment and prior malaria exposure on CogState unadjusted (raw score) outcomes were analyzed using multivariable general linear models. All analyses were performed using SAS 9.4.

## Results

### Demographic characteristics of the study participants

Additional file 1: Table S1 summarizes the characteristics of the study sample. Two hundred and thirty children were included in the study; 130 (54%) of them were male and 144 (63%) had a severe malaria episode in the past 5 years, as described in the participants section. The majority had attended school, and the highest level of education attained at the time of the CogState testing is summarized in Additional file 1: Table S1.

### Factor structure of CogState

Three factors emerged in the factor analysis of the CogState scores. The first factor comprised of the accuracy scores from the four card tasks which was labelled Processing Accuracy. The second factor comprised of speed scores also from the four card tasks and was labelled Processing Speed. The final factor had four scores from the two maze tasks (correct moves per second and total errors for both Maze Learning and Maze Chase each) which was labelled Maze Chase and Learning (Additional file 1: Table S2).

### Concurrent and convergent validity of CogState

Additional file 1: Tables S3 and S4 list the correlation coefficients between CogState and KABC-II and TOVA variables, respectively. Substantial correlations exceeding 0.30 in absolute value are highlighted below. The Groton Maze Chase Task Measuring visuomotor speed correlated with KABC-II’s Simultaneous Processing (r = 0.39, p ≤ .01) and TOVA’s Response Time (r = −0.38, p ≤ .01). One Card Learning correlated with TOVA’s Inattention (r = −0.40, p ≤ .01), Impulsivity (r = −0.41, p ≤ .01), Response Time (r = −0.42, p ≤ .01), Response Time Variability (r = −0.48, p ≤ .01) and Dʹ Prime (r = 0.53, p ≤ .01). One Back Memory correlated with KABC-II’s Visual Processing (r = 0.43, p ≤ .01) and with TOVA’s Inattention (r = −0.46, p ≤ .01), Commission Errors (r = −0.35, p ≤ .01), Response Time (r = −0.51, p ≤ .01), Response Time Variability (r = −0.47, p ≤ .01) and D′ Prime (r = 0.55, p ≤ .01). Whereas Detection Speed, our primary outcome for attention in the CogState correlated with Response Time only (r = 0.38, p ≤ .01), Detection Accuracy which was not our primary outcome correlated with five of the six TOVA scores as did One Card Learning and One Back Memory above. There were no significant correlations between Identification with any of the KABC-II or TOVA measures. Maze learning time correlated with KABC-II Learning (r = −0.19, p < .01) and Delayed Recall (r = −0.17, p = .01).

### Test–retest reliability of CogState

Moderate correlation coefficients were observed between baseline CogState scores and those performed 8 weeks later for Groton Maze Learning (r = 0.35, p < .01), Groton Maze Chase (r = 0.42, p < .01), Detection (r = 0.32, p < .01), Identification (r = 0.43, p = < .01), One Back Memory (r = 0.57, p < .01) and One Card Learning (r = 0.50, p < .01). Additional file 1: Table S5 shows these correlation coefficients and the mean and SD for each of the CogState outcomes at baseline and 8 weeks.

### Sociodemographic influences on CogState scores

From multivariable linear models, several significant associations were found between sociodemographic factors and CogState measures. The associations are summarized as coefficients from linear models and represent the effect of each factor over and above other covariates. Age was a significant predictor of all CogState outcomes listed below. The reported coefficients represent an increase (or decrease if negative) in the respective CogState score with each year of age: Correct Moves Per Second (coefficient = 0.30, standard error = 0.01, p < .01), One Card Learning Accuracy (coefficient = 0.21, standard error = 0.04, p < .01), One Card Back Accuracy (coefficient = 0.32, standard error = 0.04, p < .01), Identification Time (coefficient = −0.14, standard error = 0.05, p < .01), Detection Time (coefficient = −0.19, standard error = 0.05, p < .01), Errors in Maze Learning (coefficient = −0.12, standard error = 0.05, p = .02), and Detection Accuracy (coefficient = 0.24, standard error = 0.05, p < .01). Level of education summarized as primary school grade of 1 or higher versus no schooling or nursery was significantly related to One Card Learning Accuracy (coefficient = 0.35, standard error = 0.15, p = .02) and Errors in Maze Learning (coefficient = 0.38, standard error = 0.18, p = .03). As with age, the coefficient indicates the increase in CogState score for those in primary school versus those who are not. Finally, male sex was associated with lower CogState scores on One Card Learning Accuracy (coefficient = −0.32, standard error = 0.12, p < .01) and Card Identification Time (coefficient = −0.28, standard error = 0.14, p = .05).

## Discussion

This study set out to examine the psychometric properties of CogState in Ugandan children. CogState’s tests correlated with corresponding scores from the TOVA and KABC-II. CogState had a three factor structure with scales measuring Processing Accuracy, Processing Speed and Maze Chase and Learning. CogState scores also had low to moderate test–retest reliabilities when tests done 2 months apart were compared. CogState scores were affected by age, sex and education with older children, males and those in higher classes performing better.

Factor analysis of CogState in other studies has produced varying factor structures which is mainly due to the different tests included in the studies. Yoshida et al. [24] examined the factor structure of CogState in Japanese adults with schizophrenia. Tests included in the battery were Detection, Identification, One Back Memory, One Card Learning, Maze Learning, International Shopping List Task, Continuous Paired Associative Learning Task and the Social Emotional Cognition Task. The first factor was composed of the Continuous Paired Associative Learning Task, One Card Learning, One Back Memory and the Maze Learning Task that require memory while the second factor was composed of Detection and Identification that assess attention and psychomotor speed. The third factor was composed of the Social Emotional Cognition Task. A similar study in China that tested the validity and reliability of the Chinese language version of CogState used similar tests as Yoshida et al. [25] except the One Back Memory Test was replaced with the Two Back Memory test. A two factor solution was seen in this study with Detection and Identification loading on the first factor and the rest of the tests on the second factor.

These two studies and the present study suggest that Detection and Identification tasks consistently load on the same factor, most likely because they are both simple and more complex measures of reaction time to target stimuli. The present study’s factor structure is further categorised into accuracy or speed for the card tasks with the maze tasks loading on a separate factor for both correct moves and errors. Together these studies imply that the CogState test measure distinct abilities which are related by the unit of measurement of the outcome (e.g. speed, accuracy, correct moves or errors in the present study) or by the underlying construct being measured (e.g. memory, attention or social emotional cognition in the studies by Yoshida et al. [24] and Zhong et al. [25]). Careful thought therefore must be given to the outcome to be considered in studies, (i.e. whether speed, accuracy, errors or correct moves) as these measure different constructs.

In an earlier study among HIV infected Ugandan children, we observed correlations between all the KABC-II subtests and CogState’s Maze Learning, One Back Memory and One Card Learning [8]. The present study only had significant correlations between KABC-II’s Simultaneous Processing and CogState’s One Back Memory. Higher correlations between CogState tests and other tests measuring similar outcomes have been reported elsewhere, though theses were mainly in adults. In HIV infected adults in Uganda, a moderate correlation of 0.55 was observed between the cumulative scores of CogState and a standard neuropsychological battery [26]. The sensitivity of CogState in this study was 65% and specificity was 63%. Our study did not assess sensitivity and specificity.

Overton and colleagues assessed cognition in HIV infected adults using CogState and a battery of tests assessing attention/psychomotor speed, fine motor speed skills, learning and memory, executive functioning, fluency and set shifting/response inhibition [27]. Overton et al. did not include the maze tasks. Significant correlations were mainly observed between CogState’s attention measures (Detection and Identification) and the other battery of attention tests. These correlations were for the speed measures and not the accuracy measures of Detection and Identification which is contrary to what we observed for Detection’s speed and accuracy. We also observed correlations between CogState measures and the TOVA. One Card Learning and One Back Memory correlated with most of the TOVA tests implying a relationship between attention capacity and learning and working memory. Low inattention, impulsivity and quick reaction times are foundational to learning ability and working memory capacity. The results between our study and Overton et al. could in part be due to different samples, adults in the Overton study and children in ours.

The test–retest reliabilities of CogState tests were low to moderate in the present study, possibly due in part to a relatively long time period (8 weeks) between two assessments. Higher reliabilities have been observed elsewhere with less time between repeated assessments. Eckner et al. [28] reported an intraclass correlation coefficient of 0.51 for the Detection task among college athletes. A moderate correlation of 0.62 was observed in the Maze Learning task administered 1 month after the baseline testing among Chinese adults with schizophrenia [25]. In a study to examine practise effects when CogState is used repeatedly, children who were administered four CogState trials within 2 h, test–retest reliabilities between the first and second sessions ranged from 0.58 to 0.73 for tests similar to what we administered in the present study [29]. No practice effects were observed after the second trial suggesting stability of performance over time.

In normal adults who did four CogState sessions within 3 h, practise effects were also observed between the first and second session with minimal effects observed later [30]. A similar study had one group perform four sessions in 1 day and 1 week later and another group performed two sessions in 1 day and a month later [31]. As was seen in the earlier studies, practice effects were observed in the first two sessions in those who did four sessions in 1 day and minimal practise effects at 1 week. No effects were observed in the group that was tested at 1 month. These studies suggest that the follow-up scores might be better to use and also suggest that in these populations without much computer experience, at least one practice or warm-up test is needed.

Age and education were the main factors associated with CogState performance. Older age correlated with performance on One Card Learning, One Back Memory and Detection while a higher grade was associated with better performance in Maze Chase and One Back Memory. Education was also the main predictor of cognition in Ugandan school age children in an earlier study [21]. Other predictors were nutrition status and quality of the home environment, which were not associated with CogState scores in the present study.

This study had some limitations mainly because it was designed to answer other questions and not the validity of CogState. The design of the original study made it impossible to include all participants in the test–retest analysis. In addition, the heterogeneous sample with three different groups may have introduced some bias in the repeated assessments. Prior studies show that children with a history of severe malaria may have impaired cognitive functioning implying that performance at baseline may not always be correlated with follow-up assessments in the malaria groups [1, 19, 20]. However the short interval of 8 weeks between these tests may reduce the likelihood of this happening.

### Conclusions and recommendations

CogState has moderate test–retest reliability in Ugandan children with its Detection Accuracy being consistently correlated with attention scores from the TOVA. The three factor solution has Processing Speed, Processing Accuracy and Maze Chase and Learning as the underlying factors. As such, CogState provides a practical and efficient means of assessing several core domains of cognitive ability (working memory, attention, visual-spatial learning) in a cross-cultural context with African children. Although we documented in our study that CogState can be used to assess cognitive abilities in Ugandan children neurocognitively at risk from infectious disease (severe malaria), careful consideration has to be given to which outcome measures to assess (e.g., processing speed versus accuracy) and the extent to which these are dependent upon the integrity of more foundational core domains such as attention. CogState is a reasonably sensitive, efficient, and practical assessment of such domains for at-risk African children.

## Additional file

**Additional file 1.** Tables for participant characteristics, validity and reliability of CogState.

## Abbreviations

BCS: Blantyre Coma Score
CM: cerebral malaria
CC: community children
KABC-II: Kaufman Assessment Battery for Children, 2nd edition
SMA: severe malarial anemia
TOVA: Test of Variables of Attention
