# Supplementary material for: Validation of the CogState battery for rapid neurocognitive assessment in Ugandan school age children
Source: Child Adolesc Psychiatry Ment Health. 2015 Aug 14;9:38. doi: 10.1186/s13034-015-0063-6 (PMC4536703; doi:10.1186/s13034-015-0063-6)
Supplement: Supplementary file 1 — Additional file 1. Tables for participant characteristics, validity and reliability of CogState. [file 13034_2015_63_MOESM1_ESM.doc]

Table S1. Sociodemographic characteristics of the study participants

| **Characteristic** | **N (%)** |
| --- | --- |
| Sex, male | 130 (57) |
| Malaria exposure |  |
| Cerebral malaria | 91 (40) |
| Severe malaria anemia | 53 (23) |
| No malaria | 86 (37) |
| Child’s highest level of education attained |  |
| Never attended school | 10 (4) |
| Nursery | 121 (53) |
| P1 | 47 (20) |
| P2 | 25 (11) |
| P3 | 9 (4) |
| P4 | 9 (4) |
| P5 | 5 (2) |
| P6  P7 | 3 (1)  1 (1) |
|  |  |
|  | **Mean (St. Dev.)** |
| Age in years | 6.99 (1.67) |
| Height for age z-score | -1.00 (1.04) |
| Weight for age z-score | -0.94 (0.84) |
| Total SES score* | 9.62 (9.99) |

Table S2. Rotated factor pattern for the z-scores of CogState performance measures.

| Variable | Factor 1:  Processing Accuracy | Factor 2:  Processing Speed | Factor 3:  Maze chase and learning |
| --- | --- | --- | --- |
| CogState accuracy of card detection | **0.86** | 0.00 | 0.08 |
| CogState accuracy of card identification | **0.92** | 0.10 | 0.12 |
| CogState accuracy of one-card learning | **0.74** | 0.29 | -0.12 |
| CogState accuracy of one-back card memory | **0.79** | 0.01 | -0.16 |
|  |  |  |  |
| CogState speed of card identification | 0.27 | **0.71** | 0.17 |
| CogState speed of one-card learning | -0.20 | **0.78** | -0.19 |
| CogState speed of one-back card memory | 0.16 | **0.78** | -0.04 |
| CogState speed of card detection | **-0.54** | **0.45** | -0.05 |
|  |  |  |  |
| CogState correct moves per second, maze chase | 0.34 | -0.26 | **-0.54** |
| CogState correct moves per second, maze learning | 0.34 | -0.25 | **-0.54** |
| CogState total errors, maze chase | 0.02 | -0.17 | **0.76** |
| CogState total errors on first attempt, maze learning | 0.14 | -0.11 | **0.78** |
|  |  |  |  |

Factor loadings of 0.40 and higher are bolded.

Table S3.Concurrent and convergent validity of principal CogState performance measures with KABC global scale scores

| Variable | Sequential processing | Simultaneous processing | Learning | Planning | Delayed recall | Mental processing index |
| --- | --- | --- | --- | --- | --- | --- |
| CogState correct moves per second, maze chase | 0.06 | **0.39** | 0.22 | 0.28 | 0.18 | 0.24 |
| CogState accuracy of one- card learning | 0.12 | 0.28 | 0.19 | 0.23 | 0.05 | 0.18 |
| CogState accuracy of one-back card memory | 0.14 | **0.43** | 0.27 | 0.28 | 0.09 | 0.26 |
| CogState speed of card identification | 0.03 | -0.05 | -0.06 | 0.03 | -0.12 | -0.03 |
| CogState speed of card detection | -0.09 | -0.20 | -0.09 | -0.19 | -0.03 | -0.13 |
| CogState total errors on first attempt, maze learning | -0.02 | -0.06 | -0.19 | -0.13 | -0.17 | -0.10 |
| CogState accuracy of card detection | 0.14 | 0.27 | 0.20 | 0.21 | 0.03 | 0.19 |

Moderate correlation coefficients of .30 or higher in absolute value are bolded. Correlations exceeding 0.12 in absolute value were significant (significantly different from zero) in this table.

Table S4. Concurrent and convergent validity of principal CogState performance measures with TOVA scores

| Variable | Signal Omission errors % | Signal Commission errors % | Correct Response time | Response time variability | D’prime signal detection | ADHD index score |
| --- | --- | --- | --- | --- | --- | --- |
| CogState correct moves per second, maze chase | -0.27 | -0.17 | **-0.38** | -0.28 | **0.32** | 0.01 |
| CogState accuracy of one- card learning | **-0.40** | **-0.41** | **-0.42** | **-0.48** | **0.53** | 0.24 |
| CogState accuracy of one-back card memory | **-0.46** | **-0.35** | **-0.51** | **-0.47** | **0.55** | 0.21 |
| CogState speed of card identification | -0.05 | 0.02 | 0.10 | 0.03 | -0.02 | 0.01 |
| CogState speed of card detection | 0.21 | 0.12 | **0.38** | 0.22 | -0.18 | -0.13 |
| CogState total errors on first attempt, maze learning | 0.05 | -0.05 | 0.00 | -0.10 | 0.01 | 0.13 |
| CogState accuracy of card detection | **-0.43** | **-0.40** | **-0.50** | **-0.50** | **0.53** | 0.32 |

Moderate correlation coefficients of .30 or higher in absolute value are bolded. Correlations exceeding 0.12 in absolute value were significant (significantly different from zero) in this table.

Table S5. Descriptive statistics and correlations between pairs of principal CogState performance measures at baseline and 8 weeks for the passive control group

| Variable | Mean (St Dev), baseline | Mean (St Dev), 8 weeks | P-value for paired comparison of baseline with 8 weeks | Correlation coefficient,  baseline with 8 weeks |
| --- | --- | --- | --- | --- |
| CogState correct moves per second, maze chase | 0.11 (0.15) | 0.18 (0.18) | **<0.01** | **0.42** |
| CogState accuracy of one- card learning | 0.44 (0.20) | 0.47 (0.22) | 0.20 | **0.50** |
| CogState accuracy of one-back card memory | 0.42 (0.28) | 0.50 (0.32) | **<0.01** | **0.57** |
| CogState speed of card identification | 2.96 (0.15) | 3.03 (0.15) | **<0.01** | **0.43** |
| CogState speed of card detection | 2.87 (0.13) | 2.89 (0.10) | 0.20 | **0.32** |
| CogState total errors on first attempt, maze learning | 73.96 (66.89) | 82.13 (90.69) | 0.99 | **0.35** |
| CogState accuracy of card detection | 0.68 (0.44) | 0.81 (0.40) | **<0.01** | **0.54** |
